# The Importance of Visceral Hypersensitivity in Irritable Bowel Syndrome—Plant Metabolites in IBS Treatment

**DOI:** 10.3390/ph16101405

**Published:** 2023-10-03

**Authors:** Ewa Dudzińska, Andreas M. Grabrucker, Paweł Kwiatkowski, Robert Sitarz, Monika Sienkiewicz

**Affiliations:** 1 Department of Dietetics and Nutrition Education, Medical University of Lublin, 20-093 Lublin, Poland; 2 Department of Biological Sciences, University of Limerick, V94 PH61 Limerick, Ireland; andreas.grabrucker@ul.ie; 3 Bernal Institute, University of Limerick, V94 PH61 Limerick, Ireland; 4 Health Research Institute (HRI), University of Limerick, V94 PH61 Limerick, Ireland; 5 Department of Diagnostic Immunology, Pomeranian Medical University in Szczecin, Al. Powstancow Wlkp. 72, 70-111 Szczecin, Poland; pawel.kwiatkowski@pum.edu.pl; 6 Department of Human Anatomy, Medical University of Lublin, 20-090 Lublin, Poland; robert.sitarz@umlub.pl; 7 First Department of Surgical Oncology, St. John’s Cancer Center, 20-090 Lublin, Poland; 8 Department of Pharmaceutical Microbiology and Microbiological Diagnostic, Medical University of Lodz, Muszynskiego 1, 90-151 Lodz, Poland; monika.sienkiewicz@umed.lodz.pl

**Keywords:** visceral hypersensitivity, irritable bowel syndrome, vagus nerve, nutraceuticals

## Abstract

The visceral stimuli from the digestive tract are transmitted via afferent nerves through the spinal cord to the brain, where they are felt as pain. The overreaction observed in the brain of irritable bowel syndrome (IBS) patients may be due to increased peripheral sensitivity to stimuli from the gastrointestinal tract. Although the exact pathway is uncertain, attenuation of visceral hypersensitivity is still of interest in treating IBS. It has been shown that stress stimulates the sympathetic nervous system while inhibiting the vagus nerve (VN). In addition, stress factors lead to dysbiosis and chronic low-grade inflammation of the intestinal mucosa, which can lead to lower gastrointestinal visceral hypersensitivity. Therefore, an important goal in the treatment of IBS is the normalization of the intestinal microflora. An interesting option seems to be nutraceuticals, including Terminalia chebula, which has antibacterial and antimicrobial activity against various pathogenic Gram-positive and Gram-negative bacteria. Additionally, short-term transcutaneous vagus nerve stimulation can reduce the stress-induced increase in intestinal permeability, thereby reducing inflammation. The conducted studies also indicate a relationship between the stimulation of the vagus nerve (VN) and the activation of neuromodulatory networks in the central nervous system. Therefore, it seems reasonable to conclude that a two-way action through stimulating the VN and using nutraceuticals may become an effective therapy in treating IBS.

## 1. Introduction

Irritable bowel syndrome (IBS) is a severe problem in the health service. It is estimated that it accounts for 3% of all medical consultations. Moreover, this diagnosis is made in the case of about 40% of all outpatient gastroenterological referrals [1].

IBS is a functional and chronic gastrointestinal tract disorder characterized by recurrent bowel dysfunction associated with changes in bowel habits and abdominal discomfort or pain [2]. The epidemiology, clinical presentation, and treatment of IBS may vary in different geographical regions, primarily due to differences in diet or gastrointestinal infections, sociocultural and psychosocial factors, as well as symptom perception and reporting [3].

Patients with IBS can be categorized into four major subtypes, including IBS with prominent diarrhea (IBS-D), IBS with constipation (IBS-C), IBS with mixed symptoms of constipation and diarrhea (IBS-M), and unclassified IBS (IBS-U) [4].

No organic or macroscopic changes are found in the endoscopic examination. It is believed that various risk factors, such as mental stress, mainly in childhood, a previous transient inflammation or chronic low-grade inflammation of the gastrointestinal tract [5], abnormal intestinal flora, dysfunction of colon motility, and disorders of serotonin secretion, lead to the development of IBS symptoms [6].

Undoubtedly, the disturbance of changes in the composition of the microbiome with defects in its stability and diversity plays a significant role in developing IBS. Emerging data support the association of the microbiota with brain–gut dysfunctions such as anxiety disorders or depression-like behavior anxiety [7].

It is generally accepted that functional bowel diseases are psychogenic. The enteric nervous system (ENS) is involved in the control of gastrointestinal motility along with the interaction of central and peripheral mechanisms. The ENS is a center of integrative neuronal activity that can regulate gut motility and is involved in a bi-directional dialogue with the CNS, the so-called brain–gut axis [8].

Therefore, a better understanding of brain–gut signaling may contribute to the development of new therapies that will be helpful in the effective treatment of irritable bowel syndrome.

Treating patients with IBS is difficult and requires a personalized approach. Patient education about the disease, dietary counseling, and stress reduction are key. The therapeutic approach may include both non-pharmacological therapies and pharmacotherapy. Managing stress and changing diet and lifestyle can often control mild symptoms. However, the choice of pharmacological treatment (Table 1) is based on the dominant symptom [9].

For patients with IBS-D, therapeutic options mainly include antibiotics such as rifaximin, the nonabsorbable drug rifamycin, which has been shown to significantly reduce global IBS symptoms, bloating, and loose stools. For the treatment of loose stools, often prescribed are peripheral opioid agonists such as loperamide, mixed opioid agonists, or antagonists (eluxadoline); the bile acid sequestrants cholestyramine, colestipol, and colesevelam are also useful therapeutic options for the treatment of IBS-D [10]. The 5-hydroxytryptamine type 3 serotonin receptor antagonists alosetron, ondansetron, and ramosetron are among the drugs initially developed for treating chemotherapy-induced nausea but have also been shown to slow colonic transit time [10]. In the case of IBS-C, bulking medications and osmotic laxatives are used, while lubprostone and linaclotide should be reserved only for difficult-to-treat patients [9].

Abdominal pain is usually treated with antispasmodics such as mebeverine or hyoscine. Trimebutin, which has spasmolytic activity and non-selective agonist activity toward intestinal μ, δ, and κ opioid receptors, has been successfully used to modulate visceral sensitivity [11].

The manipulation of gastrointestinal microflora in IBS therapy is an up-and-coming area of research because it offers the potential to modulate the microbiota through prebiotics, probiotics, and synbiotics [10]. The research confirms that probiotics have anti-inflammatory effects. In preclinical studies on rodents, the potential anti-inflammatory effect was confirmed in the case of Lactobacillus reuteri, which showed inhibition of TNF-α-induced IL-8 production, and Lactobacillus casei, which can significantly reduce the release of TNF-α in ileal tissues [12].

The transplantation of fecal microflora (FMT) also seems to be a promising therapy in the treatment of IBS.

FMT is an unconventional therapy in which patients are given fecal material from healthy donors to introduce a balanced conglomerate of microorganisms. FMT has been shown to be effective in treating recurrent Clostridioides difficile infections. FMT therapy is currently being investigated as a therapeutic option for patients with IBS. However, the results of previous studies are conflicting, and further research on the effectiveness of this therapy is required [13].

Unfortunately, it is often impossible to eliminate the symptoms of the disease. In general, the effectiveness of all drugs used to treat IBS is low, and recurrence of symptoms of the disease should be expected. For these reasons, pharmacological treatment is not considered a panacea but one element of a multimodal approach to the treatment of IBS [14].

Raskov et al. emphasize that IBS is a multifactorial complex of changes in the intestinal microflora, the immune system, and gut–brain axis signaling. However, the effectiveness of currently available therapeutic methods is somewhat limited in this case [15]. Therefore, it is reasonable to search for new therapeutic methods using, for example, plant metabolites and stimulation of the vagus nerve in the treatment of IBS symptoms.

## 2. Visceral Hypersensitivity Association with IBS

The hypersensitivity of the large intestine is a characteristic feature of all IBS subtypes because the amended rectal perception in 61% of IBS patients has been documented [16].

It is now known that visceral stimuli from the digestive tract are transmitted via afferent nerves through the spinal cord to the brain, where they are felt as pain. It is unclear where the abnormalities along the visceral pathway are located. However, they were found to be responsible for the hypersensitivity seen in IBS patients. This overreaction observed in the brain of IBS patients may be due to increased peripheral sensitivity to stimuli from the gastrointestinal tract. Although the exact pathway is uncertain, attenuation of visceral hypersensitivity is still an area of interest in treating IBS [17].

Visceral hypersensitivity (VHS) plays a key role in the pathogenesis of IBS. In the clinical presentation, VHS is defined as an increased perception of mechanical stimuli applied to the intestine, manifested by pain and discomfort [18]. Patients with visceral hypersensitivity tend to have abdominal pain, mainly attributed to intraluminal retention of the gas or solid contents and mechanical stress to the gut wall [19]. Furthermore, in patients with IBS, there is an increased tendency in reports of pain and urge due to the lower colonic distension pain threshold compared to the healthy group [20]. Research by Bouin et al. aimed to determine the sensitivity, specificity, and predictive value of pain thresholds for rectal distension assessed using an electronic barostat in 86 patients with IBS, 21 with functional dyspepsia, 26 with painless constipation, 31 with various gastrointestinal conditions, and 25 healthy controls. The study showed that IBS patients had lowered pain thresholds in response to rectal distension compared to the control group and other patients with gastrointestinal diseases [21].

However, in the studies of Grabauskas et al., it was shown that colon biopsies taken from IBS-D patients contain high levels of prostaglandin E2 (PGE2), accompanied by a 2-3-fold increase in cyclooxygenase-2 (COX2) gene expression. In contrast, fecal material in the colon of IBS patients contains increased amounts of tryptase, histamine, and other bioactive substances. The study results suggest that prostaglandin E2 produced by colon mast cells activates EP2 receptors in the submucosal sensory fibers of dorsal root ganglia (DRG) neurons, which transmit nociceptive signals to the spinal cord, which is crucial for VH induction [22].

Chronic, low-grade inflammation of the intestinal mucosa, in combination with stress factors, can lead to lower gastrointestinal visceral hypersensitivity. This is confirmed by some of the studies in a group of patients with IBS, where the level of interleukin-6 in plasma positively correlated with the level of adrenocorticotropic hormone (ACTH) stimulated by corticotropin-releasing factor (CRH) [23].

Numerous reports confirm that CRH receptors mediate visceral hypersensitivity, and classical visceral hypersensitivity is exclusively induced by CRH1. However, as demonstrated by Nozu et al., CRH1-induced changes in colon function are also influenced by the CRH2 receptor through inhibitory signaling. The work also emphasized the role of the balance of CRH1 and CRH2 signaling, which determined changes in visceral sensation and colonic contractility [24].

As studies support the hypothesis that CRH signaling pathways play a vital role in the pathophysiology of IBS, colon function and visceral perception in IBS patients may be modified through peripheral administration of αhCRH, a non-selective CRH receptor antagonist. In the work of Sagami et al., CRH receptor antagonists have been shown to inhibit stress-induced bowel motor changes in IBS patients [25].

Mogilevski and colleagues have shown that short-term transcutaneous vagal stimulation in healthy individuals can reduce stress-induced (after peripheral administration of 100 µg of CRH) increases in intestinal permeability. The vagus nerve stimulation procedure was performed percutaneously using a special Digitimer DS7A and DS7AH HV stimulator placed in the auricle. Stimulation was performed for 10 min on the right side and then for 10 min on the left ear [26].

These studies confirm the protective effect of transcutaneous vagal nerve stimulation, which indicates its potential use in several chronic gastrointestinal diseases associated with intestinal inflammation, visceral hypersensitivity, or increased intestinal permeability [26].

### 2.1. The Role of the Vagus Nerve as a Modulator of the Brain–Gut Axis

“*My function’s almost anything, and vagus is my name*” [27].

The vagus nerve (VN), also called the “great wandering protector” [28], is part of the parasympathetic nervous system, is the longest cranial nerve, and is a crucial bidirectional channel between the body and the brain, mainly for maintaining homeostasis [29]. The vagus nerve is essentially a sensory nerve that informs the brain of the state of visceral organs, such as the intestines [30].

All sensory information converges in the nuclei of the VN, which is then transmitted to many brain areas. The relevant regulatory information is transmitted from these areas by the descending efferents of the vagus nerve [28].

The balance between the activity of the sympathetic and parasympathetic systems is referred to as the sympathetic–vagal balance, which is involved in regulating homeostasis. Disruption of this balance is conducive to but also reflects pathological conditions [30].

From a physiological point of view, the area of parasympathetic preganglionic neurons modulates the functions of the digestive system. It consists of afferent fibers of the vagus nerve, nucleus tractus solitarius (NTS) neurons located in the medulla oblongata, and efferent fibers originating from the dorsal motor nucleus of the vagus nerve (DMV) within the brainstem. Neural communication between the NTS and the DMV is vital. Research indicates that the presence of various neurotransmitter hormones regulates it, but also the presence or absence of transmission afferent input to the NTS [31].

VN plays a crucial role in determining brain–body interactions. Vagal activity tone is positively influenced by health, relaxation, well-being, and emotions such as empathy, while it is negatively influenced by risk factors such as morbidity and stress [27].

The multiplicity of functions of the VN causes an increase in the interest of scientists in artificial stimulation of the vagus nerve for therapeutic purposes [27].

The research indicates a relationship between vagus nerve stimulation (VNS) and the activation of neuromodulatory networks in the central nervous system. Manual or electrical techniques are used for vagal nerve stimulation [32]. The long signals of the delivered VNS increase neuronal activity in the dorsal raphe nucleus and locus coeruleus. Moreover, to change after intense stimulation, VNS provides rapid activation of neuromodulatory pathways. Vagus nerve stimulation causes immediate changes in cortical synchronization, but the effect depends on the activation of muscarinic acetylcholine receptors [33]. Muscarinic acetylcholine receptors have been divided genetically and pharmacologically into five subtypes (M1–M5). Smooth muscle tissues show heterogeneous expression of the M2 and M3 subtypes with a population of approximately 4:1 and play an essential role in intestinal contractile responses [34]. Studies indicate that IBS patients display an exaggerated muscarinic-receptor-mediated IL-6 response [35].

Chen et al. studied the effects of vagal nerve stimulation (VNS) on CRF-induced changes in serum ACTH levels in rats. The conducted analyses showed that 2 h of continuous vagus nerve stimulation inhibited CRF-induced ACTH release compared to the control group [36]. Similarly, research by Nijsen et al. suggests that endogenous CRH reduces the vagal response to conditioned fear stress in rats [37].

A study by Pellissier et al. showed that high morning vagal tone is associated with low evening cortisol levels in healthy individuals. Unfortunately, the demonstrated correlations are not observed in IBS patients, suggesting a disorder between vagal tone and cortisol levels in these patients. Additionally, research indicates that IBS patients with low vagal tone exhibit high plasma epinephrine levels as a sign of maladjusted high sympathetic activity [38].

It has been shown that stress stimulates the sympathetic nervous system while inhibiting VN [30]. There is a close relationship between exposure to stress and developing IBS symptoms, which are influenced by both autonomic and neuroendocrine responses. Studies have shown that people who experience severe stress reactions in childhood are more likely to develop IBS later in life, showing a strong link between the brain and the digestive system [39]. The consequence of reducing the activity of the vagus nerve, for example, as a result of stress, is the increased secretion and permeability of the intestines and the intensification of inflammation in the intestines. In addition, pain receptors become more sensitive, which increases the perception of visceral pain in IBS [40]. Incorrect vagus nerve tension is described in IBS and inflammatory bowel disease (IBD), which is why it seems reasonable to state that VN (VNS) stimulation may become an effective therapy in the treatment of these diseases [30].

**Figure 1:**
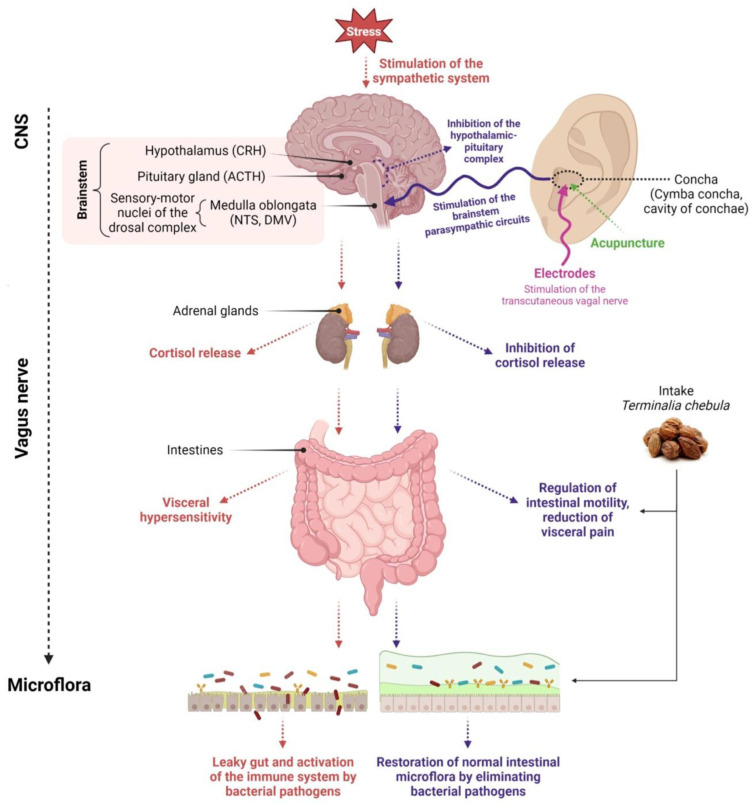
Diagram depicting the influence of various clinical methods on the restoration of microflora. Created with BioRender.com, accessed on 1 September 2023.

VNS connects specific peripheral sensors and effectors to the central nervous system. VN-mediated connections involve projections to the hypothalamus and cortex as higher brain regions, thus allowing VN modulatory access to subcortical and cortical brain regions. Thus, the signals generated in the VN can affect a wide range of basic brain functions and the whole organism’s protection (Figure 1) [33].

The external ear is the only place on the body where VN sends its only peripheral branch. The auricular branch of VN surfaces, as the afferent auricular VN (aVN), is susceptible to external stimuli in terms of peripheral nerve stimulation. It allows for easy external access via electrical stimulation in terms of aVNS, which then connects the applied stimuli to the brainstem. Thus, the auricle, especially its aVN endings, might become a critical area of the body to modulate various brain functions, offering non-invasive manipulation of the central nervous system via the vagus nerve [33].

Therefore, transcutaneous VNS (tVNS) is a very interesting therapy form. It is a non-invasive method that stimulates the afferent nerve fibers of the vagus nerve in the ear. According to the “bottom-up” mechanism of the CNS, electrical stimulation of these areas can cause changes in the activity of the VN pathway in the central structures and the brainstem. taVNS (transcutaneous auricular VNS) has been successfully used to treat disorders such as depression, epilepsy, chronic tinnitus, or pre-diabetes [41].

In recent years, transcutaneous auricular VNS (taVNS) has been reported to alleviate gastrointestinal disorders. A study of forty-two IBS-C patients randomized to a 4-week sham treatment with taVNS or taVNS showed that non-invasive taVNS reduced constipation and abdominal pain in IBS-C patients. Moreover, the applied taVNS therapy reduced serum TNF-α and IL-6 levels and plasma 5-HT levels. The improvement in IBS-C symptoms was justified by the integrative effect of taVNS on gut function mediated by autoimmune mechanisms [42]. Clinical data on the use of taVNS in treating conditions such as depression and pain suggest that it is safe and well tolerated [32].

Both neural communication via the vagus nerve and hormonal communication via the hypothalamic–pituitary–adrenal axis (HPA axis) together enable the brain to influence the activity of functional effector cells of the intestine, such as epithelial cells, immune cells, intestinal neurons, smooth muscle cells, and enterochromaffin cells. It turns out that these cells are under the influence of the intestinal microflora. The gut microbiota also significantly impacts the brain–gut axis, locally interacting with intestinal cells and the enteric nervous system (ENS) and directly affecting the metabolic and neuroendocrine systems [43].

Acupuncture is also a form of therapy for IBS. It is a traditional Chinese medical practice that is increasingly accepted and used in Western society. Acupuncture, widely used in IBS, involves stimulation of the vagus nerve and, thus, stimulation of the somatic nervous system. Studies show that the applied therapy can alleviate visceral hypersensitivity and improve intestinal motility in patients with IBS [44].

A multicenter, randomized, controlled trial involving 531 patients diagnosed with IBS showed that acupuncture was more effective than pinaverium bromide in relieving IBS symptoms and improving quality of life during a 1.5-month treatment and a subsequent three-month follow-up period [45].

### 2.2. The Vagus Nerve Can Recognize the Microflora and Provide This Information to the Central Nervous System

Changes in the composition of the intestinal flora are important in developing intestinal diseases. Unfortunately, the mechanisms leading to the development of the disease process remain largely undefined. In this case, the activity of signaling molecules and the recognition of bacterial epitopes by immune cells of both the intestinal epithelium and the mucosa may play an important role [46].

Stress can modify the intestinal microflora by releasing stress hormones, while dysbiosis is observed in both IBS and IBD. Communication between the brain and the microflora is two-way, through many trials: nervous via VN and/or spinal cord, hormonal, immunological, and metabolic [30].

Dysbiosis of the intestinal microbiota composition may cause intestinal permeability, intestinal motility disorders, visceral hypersensitivity, and anxiety disorders, or depression [47].

Commensal gut bacteria and gut pathogens can also alter and modulate visceral hypersensitivity to pain. The microbiota mediates visceral nociception by regulating visceral afferents either directly or through interfering with gut barrier function and the gut immune system [48]. In preclinical studies in animal models, the gut microbiota has been found to play a crucial role in hypersensitivity. For example, antibiotics administered early in life have been shown to cause long-term exacerbation of visceral pain in adult mice by disrupting the gut microbiota. In contrast, studies in rats showed that treatment with vancomycin early in life also increased visceral pain caused by distension of the large intestine. Other preclinical studies have also provided evidence that probiotics can regulate visceral pain. Data from studies in microbe-free mice showed that the gut microbiota has a decisive influence on visceral pain, as germ-free mice exhibited visceral hypersensitivity accompanied by upregulation of Toll-like receptors and cytokines in the spinal cord.

In contrast, hypersensitivity was abolished through colonization by postnatal microflora from conventionally colonized animals [49]. A clinical trial study of 362 IBS women showed that *B. infantis* effectively reduces pain and abdominal bloating and improves intestinal movements after four weeks of treatment compared to placebo. Similarly, the use of *L. rhamnosus* and *L. plantarum* in patients with IBS showed reduced pain and bloating in two studies. In another study, where patients were administered probiotics for eight weeks, abdominal pain was also reduced [48].

Improper fermentation can be an important factor in the development of diseases of the digestive tract. Intestinal infections or antibiotic therapy can damage the colonic microflora, increasing fermentation and gas accumulation. Intestinal gas is associated with symptoms such as bloating, discomfort, constipation, belching, and abdominal pain. In addition, they are among the most common health complaints that cause people to visit a gastroenterologist [50].

Sulfate-reducing bacteria (SRB) present in the human colon dissimilate sulfate to hydrogen sulfide in a process called sulfate dissimilation reduction. Hydrogen sulfide is highly toxic not only to the host but also to the bacteria that produce it. High concentrations of hydrogen sulfide lead to the inhibition of SRB growth and the development of inflammatory damage to the intestinal epithelium [51].

A diet rich in sulfate ions, such as foods preserved with sulfur oxides, increases the hydrogen sulfide concentration produced by SRBs and can lead to gastrointestinal diseases. Research reports that the products included in the Western diet contain as much as 16.6 mmol of sulfates per day [51].

SRB can exacerbate gastrointestinal disease by producing the toxic product hydrogen sulfide and reducing the production of beneficial butyrate, the preferred energy source for colonocytes [52]. Butyrate has a normalizing effect on functional disorders of the intestines and has anti-inflammatory properties in the large intestine [38]. In addition, some SRB species may be associated with gastrointestinal diseases. The incidence of Desulfovibrio piger was significantly higher in patients with inflammatory bowel disease compared to healthy controls [52]. In addition, Desulfovibrio metabolites are also crucial factors leading to the formation and development of intestinal diseases, stimulating the body to release inflammatory factors such as Interleukin-6 or Interleukin-8. One of the primary metabolites of Desulfovibrio is the hydrogen sulfide mentioned above, which is cytotoxic in high concentrations [53].

The intestinal microflora also contains methanogenic archaea.

Methanogens are essential components of the intestinal microbiota colonizing the intestines, a group of microorganisms classified as archaea. Archaea produce methane by utilizing molecular hydrogen and carbon dioxide during the oxidation of organic acids [54].

Several lines of evidence indicate that archaea are absent in infancy while ubiquitous in school-aged children, suggesting that colonization is due to childhood environmental exposure [55].

Anaerobic methanogens are the primary sources of methane in the intestines. *M. smithii*, isolated from various phylotypes, is involved in over 90% of methane production and constitutes 10% of the intestinal microbiome [56].

It has been shown that IBS-C patients have significantly different gut microbiota compared to IBS-D patients. The gut microbiome of IBS-C individuals has more archaeal methanogens than IBS-D, which produce methane by fermenting endogenous and exogenous carbohydrates [56]. The association of irritable bowel syndrome with methane makes it the center of attention and further underscores the importance of studying this relationship for disease symptoms and possible treatments for IBS.

Kim et al. investigated the importance of *M. smithii* as a determinant of methane production in IBS patients with detectable methane on a breath test and in patients without detectable methane. The studies were conducted in stool samples using quantitative polymerase chain reaction. It has been shown that *M. smithii* is the significant methanogen responsible for respiratory methane in people with IBS. In addition, the level of *M. smithii* in fecal samples correlated with the amount of methane produced in the breath test, suggesting that *M. smithii* may be the main methanogen responsible for the methane detected in human breath tests [54].

In the studies conducted by Ghoshal et al., it was found that the number of copies of *M. smithii* was higher among IBS patients, especially IBS-C, than with IBS-D and the control group [57].

Activation of the hypothalamic–pituitary–adrenal (HPA) axis by stress causes the release of CRH from the paraventricular nucleus of the hypothalamus, followed by the release of ACTH from the anterior pituitary. ACTH stimulates the adrenal cortex to release cortisol, which inhibits the vagal response.

The vagus nerve is typically stimulated in its anatomical region of the ear, i.e., in the cymba concha and cavity of concha. The most popular non-invasive forms of vagus nerve stimulation are percutaneous methods by applying stimulating electrodes on the ear skin or minimally invasive methods such as acupuncture.

Stimulation aims to transmit an impulse to the area of NTS and DMV sensory-motor nuclei of the dorsal vagal complex and transfer information from the NTS to the DMV. Stimulation of the vagus nerve in the Concha area alters the vagus nerve’s functional output and activation of the parasympathetic system.

The vagus nerve recognizes the intestinal microflora and transmits this information to the central nervous system. Stimulation of the vagus nerve through non-invasive or minimally invasive methods (acupuncture) leads to restoring the balance of the intestinal microflora. Additionally, the balance of the intestinal microflora is restored through oral intake of nutraceuticals. Created with Biorender.com accessed on 1 September 2023.

### 2.3. The Role of Serotonin in IBS

Numerous studies confirm that serotonin plays a key role in intestinal neurotransmission, initiation and propagation of gastrointestinal motor activity, and gut–brain signaling [8].

Other studies report that the hormone serotonin 5-hydroxytryptamine (5-HT) may also modulate visceral perception. Serotonin in the intestine is released from cells (EC). It is believed that the 5-HT3 and 5-HT4 receptors may play an essential role in transmitting visceral sensation from the gut [58]. It is well known that 95% of the organism’s serotonin is produced in the gut, where it has been increasingly examined for its paracrine, autocrine, and hormonal effects [59].

The work of Bulbring et al. highlighted for the first time the link between 5-HT, the peristaltic movement, and the stimulation of peristaltic waves. In isolated preparations of the guinea pig ileum, it was shown that synthetic intestinal 5-HT instigates the peristaltic motion and, further, that stimulation of the peristaltic movement causes 5-HT to be secreted in the gut [59]. In connection with the above, there must be a strong relationship between IBS pathogenesis (5-HT) and the serotonin transporter (SERT) [60]. As a sodium neurotransmitter and symporter family member, SERT regulates the extracellular availability of 5-HT in the intestine through 5-HT uptake. Genetic or environmental abnormality of SERT expression is associated with abnormal 5-HT levels in the intestinal mucosa and may contribute to the development of functional gastrointestinal diseases such as IBS [61]. 5-hydroxytryptamine (5-HT) is a messenger for intestinal motility, visceral sensation, and fluid secretion. SERT is also a transmembrane transporter with a high affinity for serotonin and plays a vital role in its metabolism. High serotonin levels are often associated with reduced SERT levels in IBS patients. Several studies found that SERT polymorphisms were closely associated with IBS risk in Caucasians and Asians whose serotonin levels in rectal mucosa biopsies were high, particularly in IBS-D [60].

Interestingly, an excess of serotonin in the gut may result from a protective response to infection, facilitating the rapid clearance of infecting organisms from the gut. One prospective study showed that people who developed PI-IBS after infection with C. jejuni had a significant increase in both mucosal CD3-positive lymphocytes and EC cells compared to controls [1].

Animal studies have shown that SERT knockout mice had diarrhea associated with colon hypermotility, resulting in increased water excretion in the feces [62]. Wang et al. indicated that SERT polymorphisms may contribute to the development of IBS [63]. The research so far focuses on genetic polymorphisms related to the etiology of IBS, such as SLC6A4, 5-HTTLPR, or a variable number of tandem repeats STin2 [64].

### 2.4. Plant Metabolites in IBS Treatment

**Figure 2:**
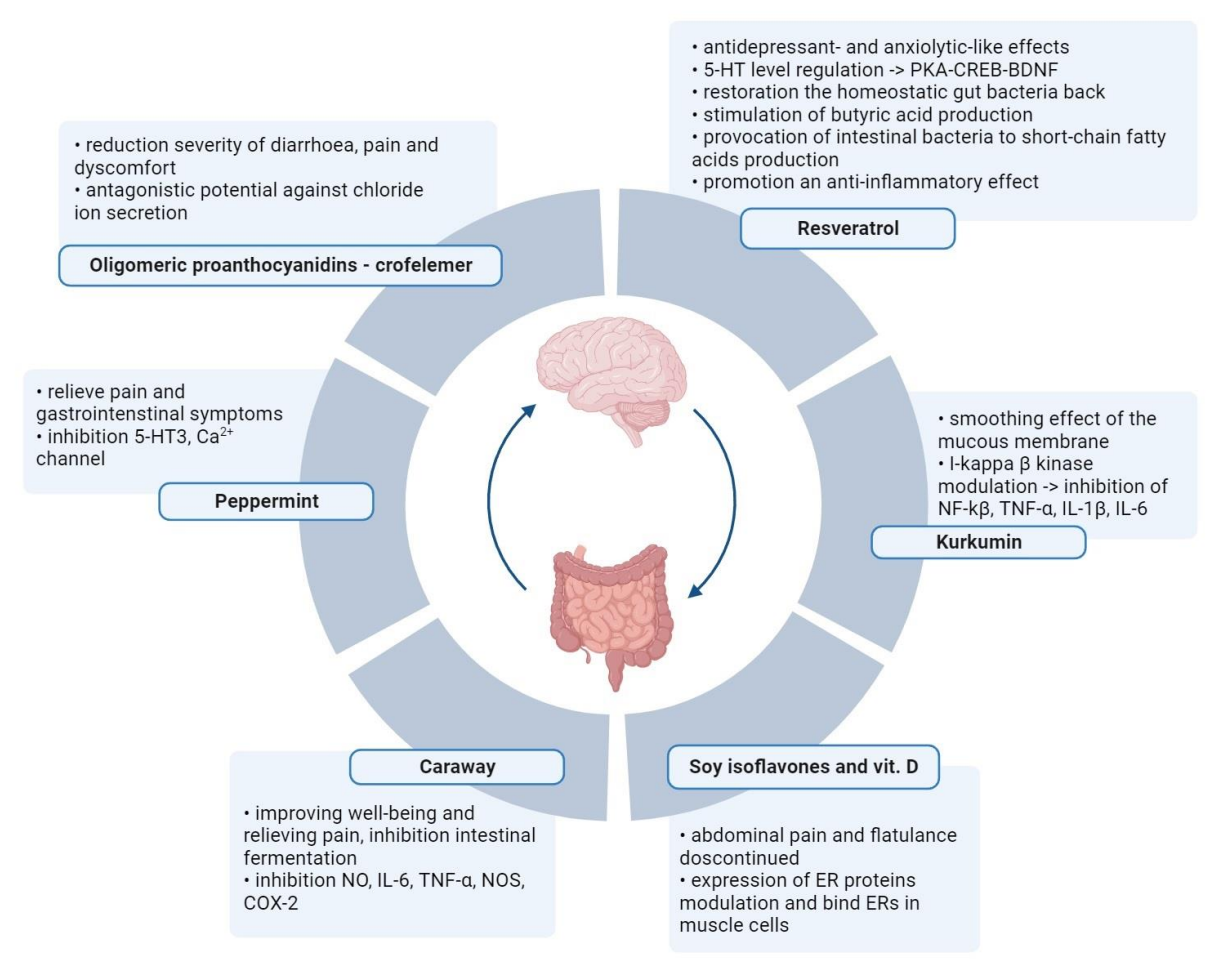
Alternative therapies in IBS with the use of active metabolites of plants. Normalization of the intestinal microflora and elimination of intestinal pathogens through oral intake of nutraceuticals. Created with BioRender.com, accessed on 1 September 2023.

Plant metabolites are used to reduce the severity of IBS symptoms (Table 2 and Figure 2). Several scientific publications describe the excellent performance of soya isoflavones and vitamin D, which allow for a reduction in plasma inflammatory markers and fecal protease activity in women with IBS [65,66]. Women between 18 and 75 years old with IBS were included in the study. Participants were randomly divided into four groups of 25 women each (placebo of vitamin D and placebo of soy isoflavones, placebo of vitamin D and soy isoflavones, vitamin D and placebo of soy isoflavones, and vitamin D and soy isoflavones) and the therapy was administered for 6 weeks. A validated IBS symptoms severity score (IBS-SSS) questionnaire and a validated IBS quality of life (IBS-QOL) questionnaire were used to verify the results. The authors reported that the effect of isoflavones persisted even after supplementation against abdominal pain and flatulence was discontinued. The soy phytoestrogens and vitamin D bind to ERs in smooth muscle cells and modulate the expression of ER proteins. Vitamin D ensures homeostasis of the intestinal mucosal barrier, and vitamin D deficiency is related to severe IBS. A significant reduction in TNF-α in the “soy with vitamin D” and “soy” groups was also found compared to the placebo group. The leukocyte nuclear factor-κβ level NF-κβ levels and fecal protease activity were significantly lower in all three treatment groups.

According to a clinical trial described by Nee et al., one plant metabolite effective in treating pain in women with IBS-D is oligomeric proanthocyanidins—crofelemer, obtained from *Croton lechleri*. Crofelemer has antagonistic potential against chloride ion secretion by the calcium-dependent chloride channel (CaCC) in the intestine and thus may help treat diarrhea and related symptoms. In a multicenter, phase II, randomized, double-blind, placebo-controlled trial of 237 women with IBS-D, 125 mg of crofelemer orally or matched placebo were tested for 2 weeks twice daily. It was shown that there was a reduction in the severity of diarrhea, pain, and discomfort, specifically in women with IBS-D. Research suggests that crofelemer is also a well-tolerated drug treatment for pain in patients with IBS-D [67].

According to studies by Portincasa et al., a combination of curcumin and fennel essential oil because of their anti-inflammatory and antispasmodic properties may be used against patients with mild-to-moderate symptoms of IBS-SSS (IBS-severity scoring system). Curcumin has a soothing effect on the mucous membrane through the modulation of I-kappa B kinase activity, driven by inhibition of nuclear factor-κB (NF-kB) and proinflammatory cytokines—tumor necrosis factor alfa (TNF-α), and interleukin 1β and 6 (IL-1 and IL-6, respectively). Anethole, chemically similar to the neurotransmitter dopamine, has a relaxant effect on intestinal smooth muscle. A randomized, double-blind, placebo-controlled, multicenter trial of 77 women and 44 men was conducted. Participants took two capsules containing curcumin (42 mg) and fennel essential oil (25 mg) or a placebo for 30 days. It was shown that the fennel reduced abdominal pain through relaxation of intestinal smooth muscle. A combination of curcumin and fennel oil, which increased the bioavailability of curcumin, was well tolerated and induced symptom relief in patients with IBS, significantly reducing symptoms and improving quality of life in IBS patients over 30 days [68].

Mahboudi reviewed randomized clinical trials that evaluated the efficacy and safety of caraway and peppermint oils for treating IBS [69]. The antimicrobial activity of caraway oil, containing mainly D-carvone and L-limonene, inhibits the intestinal fermentation processes and inhibits the formation of foam in the stomach, improving well-being and relieving pain. The effect of caraway oil consists, among other things, of inhibition of the release of nitric oxide (NO), nitric oxide synthase (NOS), cyclooxygenase-2 (COX-2), interleukin 6 (IL-6), and tumor necrosis factor-alpha (TNF-α). Menthol contained in peppermint oil has similar properties. Peppermint oil also has an antispasmodic, antidepressant effect by inhibiting human serotonergic receptors (5-HT3), blocking the Ca^2+^ channels, and leading to the relaxation of intestinal smoothness. The analysis conducted by Alammar et al. against twelve randomized trials with 835 patients provided indisputable evidence of peppermint oil’s effectiveness in treating patients with functional dyspepsia-concomitant IBS [70]. So, it was proven that treatment with the use of peppermint oil is successful in relieving pain as well as gastrointestinal and other severe or unbearable symptoms.

Resveratrol is one of the polyphenol compounds described as an effective ingredient against IBS. Intestinal dysfunction with abdominal pain and discomfort in IBS is also comorbid with emotional disorders like depression and anxiety. Yu et al. showed that resveratrol exhibits antidepressant and anxiolytic-like effects in a rat model of chronic, acute combining stress (CACS) intestinal motility disorder and visceral hypersensitivity. CACS is connected with reducing 5-hydroxytryptamine (5-HT) levels in the hippocampus and increased 5-HT expression in the gut (ileum and colon). Male Sprague-Dawley rats weighing 180–220 g were treated with resveratrol, fluoxetine, or diazepam for 1 h or 30 min before CACS for 22 days. According to their research, resveratrol has good potential for the regulation of 5-HT1A-dependence in the brain–gut axis: cAMP-dependent protein kinase, cAMP response element-binding protein, and brain-derived neurotrophic factor (PKA-CREB-BDNF), thereby contributing to alleviating conditions of depression, anxiety, visceral hypersensitivity, and intestinal motility disturbances [71]. IBD may also result from dysbiosis. Alrafas et al. tested the efficacy of resveratrol in an in vivo (2,4,6-trinitrobenzene sulfonic acid) TNBS-induced colitis mouse model using female BALB/c mice. The study showed that the resveratrol treatment reversed microbial dysbiosis. Moreover, it turned out that this compound stimulates the production of butyric acid, which has potent anti-inflammatory properties, and provokes intestinal bacteria to produce short-chain fatty acids that promote an anti-inflammatory effect by inducing Treg/IL-10 and inhibiting the inflammatory (Th1/Th17) T cell response [72].

### 2.5. Terminalia chebula and Its Potential Role in IBS Therapy

Preclinical studies indicate that the administration of a raw extract of Terminalia chebula has, among others, antispasmodic, immunosuppressive, cardiotonic, antioxidant, antimutagenic, antihepatotoxic, antimicrobial, antifungal, cytoprotective, and antidiabetic effects [73].

Numerous phytochemical reports also indicate the anxiolytic, antidepressant, sedative-hypnotic, and antioxidant effects of Terminalia chebula, which is also important in treating IBS [6].

Fruits of *Terminalia chebula* are a rich source of tannins such as terchebin, chebulinic acid, neochebulinic acid, punicalagin, ellagic acid, corilagin, terflavin A, chebulic acid, chebulagic acid, and gallic acid. These fruits additionally contain flavonoids [74].

*Terminalia chebula* has a wide therapeutic application in traditional medicine, some of which have been confirmed by modern medicine [75].

The fruit should not be finely powdered but should rather be partially crushed. The fruit is more effective when soaked in water than as a decoction or powder. The therapeutic use is mainly of Terminalia chebula fruits, which are whole fruit preparations (powdered or crushed whole fruit) for internal and external use. The daily dose ranges from 3 to 9 g [75].

In traditional medicine of the Far East, dried fruits of Terminalia chebula are successfully used in the treatment of diarrhea due to the high content of tannins, while soaked fruits are used as a laxative due to the content of anthraquinone derivatives [75]. The significant anti-diarrheal effect of the nutraceutical has been attributed to the inhibition of gastrointestinal motility and fluid secretion [6]. On the other hand, Terminalia chebula improves digestion, relieves constipation, and also reduces intestinal cramps. The studies using Terminalia chebula prove that the long-term treatment (45 days) in the study group decreased visceral pain and bloating. Moreover, no side-effects in the treated group were reported [74].

Chebulic acid has antidepressant and anxiolytic properties. In a mouse model, *Terminalia chebula* extract significantly lowered serum cortisol levels and increased monoamine neurotransmitters such as 5-hydroxytryptamine (5-HT), dopamine, and norepinephrine in brain tissues. Another gene expression study showed that cAMP response element binding protein, brain-derived neurotrophic factor, 5-HT1A, and GABAA were upregulated by tannin-rich Terminalia chebula extract [6].

Such properties of the nutraceutical can be successfully used in the treatment of IBS patients.

Several studies indicate an antibacterial effect of the antimicrobial activity of *Terminalia chebula* against various pathogenic Gram-positive and Gram-negative bacteria. *Terminalia chebula* shows comparable antibacterial activity to traditional antibiotics against intestinal pathogens such as *Escherichia coli*, *Salmonella* sp., *Shigella* sp., and *Vibrio cholerae*. In addition, ethanedioic acid isolated from the butanol fraction of *Terminalia chebula* extract has a strong inhibitory effect against *Clostridium perfringens*. On the other hand, ellagic acid has a strong inhibitory effect on *C. perfringens*. There are also reports that methyl gallate obtained from *Terminalia chebula* can be used as a potential antibacterial agent in treating infections caused by multidrug-resistant Shigella spp., which are strains showing reduced susceptibility to most recommended antibiotics [76].

## 3. Conclusions

We know more and more about the causes of IBS and its relationship with stress, anxiety, and intestinal dysbiosis. Unfortunately, it is difficult to eliminate many unfavorable factors affecting diseases in our lives. Various techniques and medicinal substances have been used for a long time, but now, thanks to the development of science, we can precisely prove their effectiveness. Scientists provide evidence of the effectiveness of non-invasive, natural methods of treating and preventing the development of IBS. It is proposed that vagus nerve stimulation by the auricle, especially its aVN endings, offers non-invasive manipulation of the central nervous system via the vagus nerve. It is also known that many synthetic drugs are analogs of plant metabolites. We also find many plant metabolites with proven anti-IBS activity. Among them are resveratrol, soya isoflavones, curcumin, fennel, caraway, and peppermint. A very interesting proposition is *Terminalia chebula* with multidirectional action as an anxiolytic, antidepressant, sedative-hypnotic, and antioxidant. This raw material improves digestion and relieves constipation, reduces intestinal cramps, decreases visceral pain, and may affect the regulation of the intestinal microbiota.

Therefore, here, we offer a comprehensive approach to IBS treatment. The proposed plant metabolites and the improvement in the gut–brain–gut axis by stimulating the vagus nerve can reduce visceral pain and thus significantly improve the lives of patients from IBS.

## Figures and Tables

**Table 1:** The main directions of conventional therapy in IBS.

Type	Medicines	Effects	Study Reference
IBS-D	Antibiotics: rifamycin and rifaximin—semisynthetic rifamycin with a broad spectrum of antibacterial activity (Gram-positive and Gram-negative bacteria, both aerobes and anaerobes).	significantly reduce global IBS symptoms, bloating, and loose stools.	[10]
	Peripheral opioid agonists: loperamide; Mixed opioid agonists or antagonists: eluxadoline; Sequestrants: cholestyramine, colestipol, and colesevelam.	loose stools	
	5-Hydroxytryptamine type 3 serotonin receptor antagonists: alosetron, ondansetron, and ramosetron.	slow colonic transit time	
IBS-C	Medications and osmotic laxatives (lubiprostone and linaclotide for difficult-to-treat patients).	laxative	[9]
General in IBS	Antispasmodics: mebeverine and hyoscine.	abdominal pain	[11]
	Spasmolytic activity and non-selective agonist activity toward intestinal μ, δ, and κ opioid receptors: trimebutins.	modulate visceral sensitivity	
	Prebiotics, probiotics, and synbiotics.	Manipulation of gastrointestinal microflora	[10]

**Table 2:** The effects of chosen plant metabolites in the treatment of IBD in clinical and pre-clinical investigations.

Therapy with the Use of Plant Metabolites	Participants/Model Organism	Effects	Study References
vitamin D and soy isoflavones	women with IBS	Abdominal pain and flatulence were discontinued; Improvement in quality of life; Reduction in the level of TNF-α, leukocyte nuclear factor-κβ, and fecal protease activity.	[65,66]
oligomeric proanthocyanidins—crofelemer	women with IBS-D	Reduction in the severity of diarrhea, pain, and discomfort.	[67]
curcumin and fennel essential oil	women and men with IBS	Reduction in abdominal pain and all the other symptoms; improved quality of life.	[68]
caraway and peppermint oil	women and men with IBS	Reduction in pain intensity and frequency and in flatulence and dyspeptic discomfort score, and sensation of pressure and fullness.	[69,70]
resveratrol	male Sprague-Dawley rats with chronic, acute combined stress	Alleviating conditions of depression, anxiety, visceral hypersensitivity, and intestinal motility disturbances.	[71]
	female BALB/c mice with TNBS-induced colitis	Restoration of homeostatic gut bacteria, and stimulation of butyric acid production; Provocation of intestinal bacteria to generate short-chain fatty acids with anti-inflammatory effect.	[72]

## Data Availability

Not applicable.
